# Supplementary figures and images for: Presynaptic PTPσ regulates postsynaptic NMDA receptor function through direct adhesion-independent mechanisms
Source: eLife. 2020 Mar 6;9:e54224. doi: 10.7554/eLife.54224 (PMC7069723; doi:10.7554/eLife.54224)

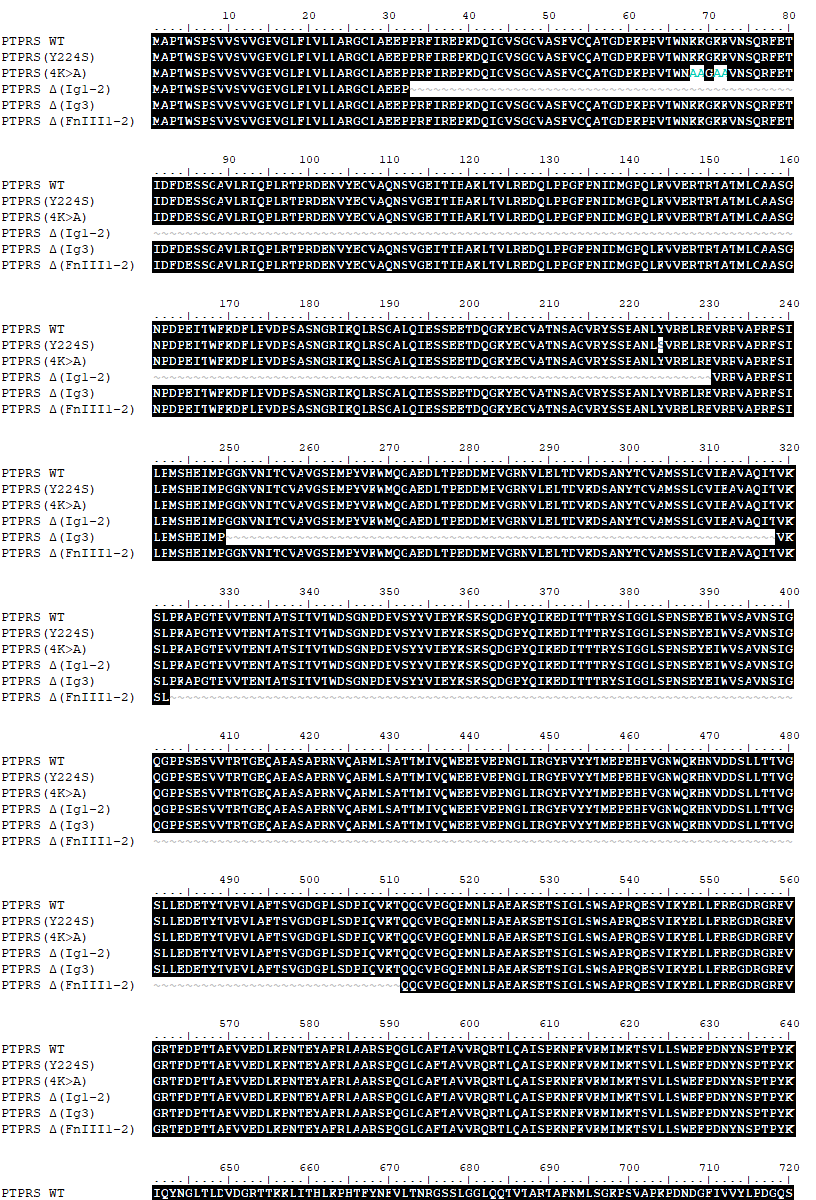

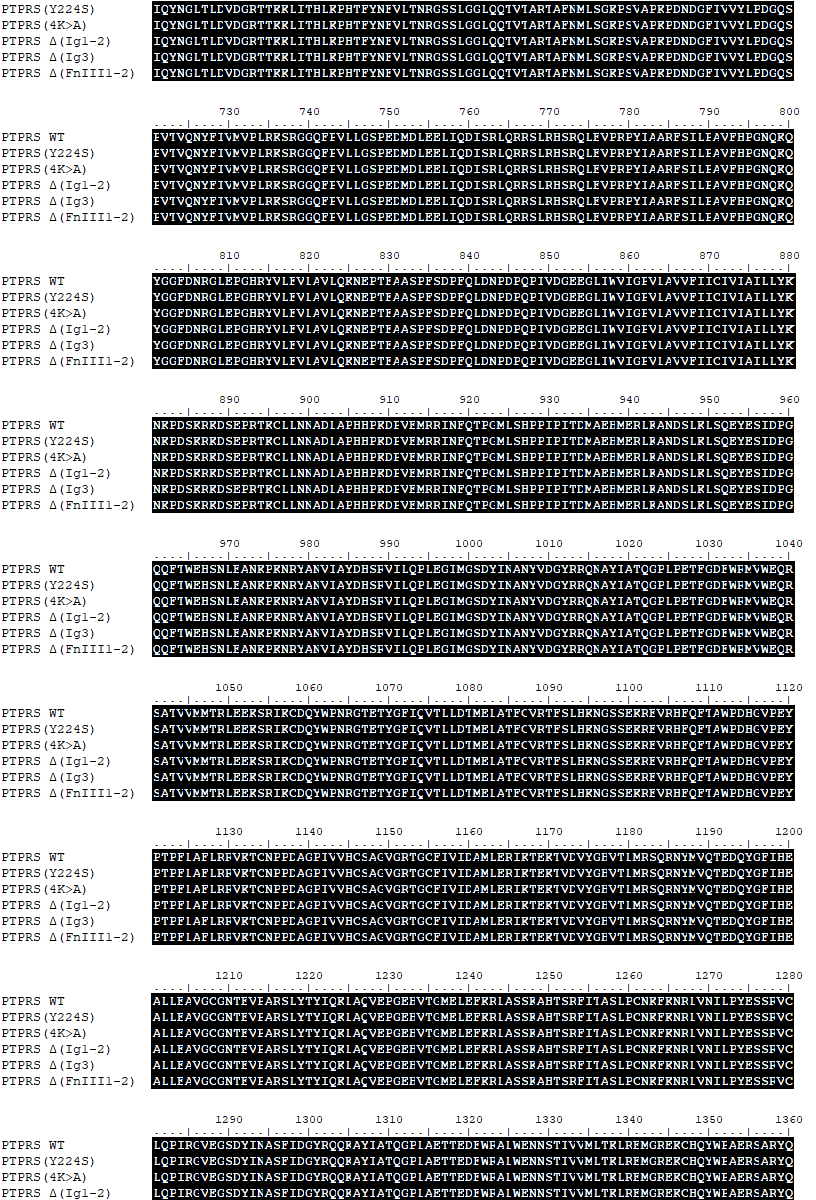

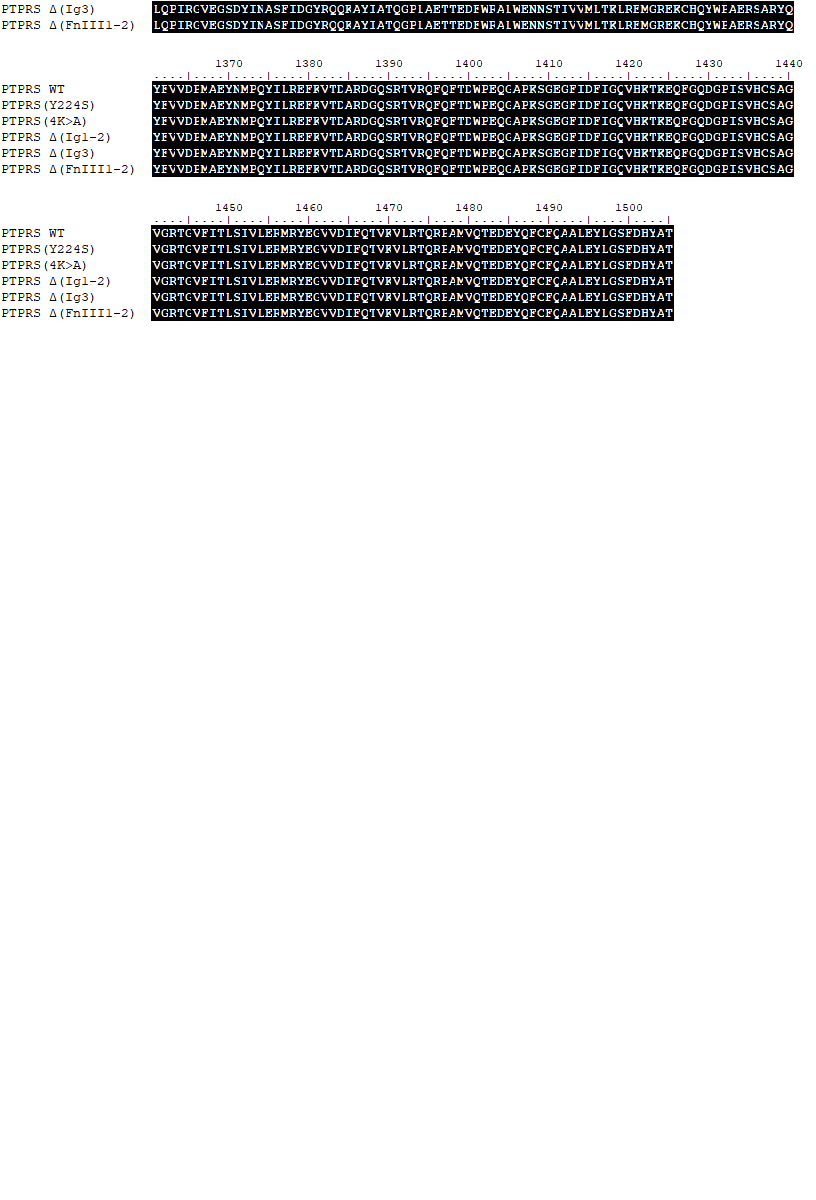

Supplement: Supplementary file 1. [file elife-54224-supp1.docx]
