# Supplementary material for: Presynaptic PTPσ regulates postsynaptic NMDA receptor function through direct adhesion-independent mechanisms
Source: eLife. 2020 Mar 6;9:e54224. doi: 10.7554/eLife.54224 (PMC7069723; doi:10.7554/eLife.54224)
Supplement: Transparent reporting form [file elife-54224-transrepform.docx]

***eLife’s* transparent reporting form**

We encourage authors to provide detailed information *within their submission* to facilitate the interpretation and replication of experiments. Authors can upload supporting documentation to indicate the use of appropriate reporting guidelines for health-related research (see [EQUATOR Network](http://www.equator-network.org/%20)), life science research (see the [BioSharing Information Resource](https://biosharing.org/)), or the [ARRIVE guidelines](http://www.plosbiology.org/article/info:doi/10.1371/journal.pbio.1000412) for reporting work involving animal research. Where applicable, authors should refer to any relevant reporting standards documents in this form.

If you have any questions, please consult our Journal Policies and/or contact us: [editorial@elifesciences.org](mailto:editorial@elifesciences.org).

**Sample-size estimation**

Throughout this study, 1) electrophysiological data, 2) animal behavior data, 3) protein analysis data, and 4) proteomic data were analyzed to get a conclusion. During this processes, we did not used any explicit power analysis to designate the appropriate sample size in all types of experiments. Rather than using power analysis, we referred diverse literatures in this field and used generally accepting number of animal/cells. The reasons we did not using power analysis experiments by experiments are following:

For the 1) electrophysiological data, experiments in Figure 1 is conducted first and followed by experiments in Figure 2-4. During field recording remarked in Figure 1 (Except for Figure 1A-B), raw data is harvested by one researcher and quantified by the independent well-trained researcher. In this processes, it would be better to harvest data within the short time window, when we considering animal availability (ages, gender, and genotypes), reducing variation, and experimenter time-scheduling. During the experiment, minimum slice numbers are decided at least 10 by each group, except for crystal-clear results (i.e. TBS-LTP in Figure 1F; used 8 by each group). We adapted similar animal/slices number in data of Figure 2-4.

For the 2) animal behavior data, each experiments were conducted through at least 2 cohorts (up to 4). Each animal number in a cohort was decided by the littermate population size, age, and gender. We designated a minimum number of animal as 10 by each group at the first, and followed. It might be larger (but not smaller) by the cohort size.

For the 3) protein analysis data, and 4) proteomics data, at least standard is 3 by the group in literatures. We used at least 5 animals for each analysis to confirm and validate it. For SPM/PSD/proteomics samples, 2 brains pooled in the one sample (3/3 number means 6/6 mice).

**Replicates**

For the electrophysiological test, it only biologically replicated with appropriate number of animal (at least 8-10, by the groups), without technically replication.

For the behavior test, it biologically replicated with at least 2 cohorts.

For the protein analysis, it biologically replicated with at least 3 samples (from more than 5 mice) by the group, and technically replicated with at least 3 times with the same sample.

For the proteomics, it does not replicated.

Replicates information did not mentioned in the submission but the criteria is written in material and methods section.

**Statistical reporting**

About mentioning below, you can also refer in material and method section. And detailed statistical information is attached in S2_Statistical Table.

For the two-column test, we firstly adapted normality test by D’Agostino and Pearson test and Shapiro-Wilk test. In case of any of column in any of test were significantly not normal, we used Mann-Whitney test, else, we used Unpaired Student’s t-test. In case of adapting Student’s t-test, considering the result of variation discrepancy test, we adapted Welch’s correction when variations of each two columns is significantly different. All of abovementioned test is adapted in two-tailed manner.

For experiments with the smaller number of samples, we conducted One-sample t-test to reduce lane-by-lane difference.

For the n-column test with a single variable, we adapted 1-WAY ANOVA with Dunnett’s multiple comparison test.

For tests more than two variable, we used 2-WAY ANOVA repeated measure, with Sidak’s multiple comparison test. The multiple comparison test results were used to analyze only when any of single factor or interaction has significant impact.

All of abovementioned statistical test were conducted in GraphPad Prism 7. And each of N number, and statistical methodology is referred in the each Figure legend. And noted by the SEM in Figure itself.

For statistical analysis of Proteomic data, raw data and statistical procedure is attached in S1_PTM-Scan molecule list. For DAVID-analysis, q-value evaluates the importance of each GO terms. To check methodology, please cite <http://david.ncifcrf.gov> page.

**Group allocation**

- Indicate how samples were allocated into experimental groups (in the case of clinical studies, please specify allocation to treatment method); if randomization was used, please also state if restricted randomization was applied
- Indicate if masking was used during group allocation, data collection and/or data analysis

Please outline where this information can be found within the submission (e.g., sections or figure legends), or explain why this information doesn’t apply to your submission:

No group allocation was adapted in this study.

**Additional data files (“source data”)**

- We encourage you to upload relevant additional data files, such as numerical data that are represented as a graph in a figure, or as a summary table
- Where provided, these should be in the most useful format, and they can be uploaded as “Source data” files linked to a main figure or table
- Include model definition files including the full list of parameters used
- Include code used for data analysis (e.g., R, MatLab)
- Avoid stating that data files are “available upon request”

Please indicate the figures or tables for which source data files have been provided:

No code, no meta-data, and numerical data were used in this study, raw data of proteomics is attached in Supplementary file 2_PTM-Scan molecule list.
